# Family Adjustment When Facing Pediatric Cancer: The Role of Parental Psychological Flexibility, Dyadic Coping, and Network Support

**DOI:** 10.3389/fpsyg.2019.02740

**Published:** 2019-12-10

**Authors:** Marieke Van Schoors, Annick Lena De Paepe, Jurgen Lemiere, Ann Morez, Koenraad Norga, Karolien Lambrecht, Liesbet Goubert, Lesley L. Verhofstadt

**Affiliations:** ^1^Department of Experimental Clinical and Health Psychology, Ghent University, Ghent, Belgium; ^2^Department of Pediatric Hemato-Oncology, University Hospitals Leuven, Leuven, Belgium; ^3^KU Leuven, Leuven, Belgium; ^4^Department of Pediatric Hemato-Oncology and Stem Cell Transplantation, Ghent University Hospital, Ghent, Belgium; ^5^Department of Pediatric Oncology, Antwerp University Hospital, Antwerp, Belgium; ^6^University of Antwerp, Antwerp, Belgium; ^7^Department of Pediatric Hemato-Oncology and Immunology, University Hospital Brussels, Brussels, Belgium

**Keywords:** pediatric cancer, family, parents, psychological flexibility, dyadic coping, network support

## Abstract

**Objectives:**

Pediatric cancer is a life-threatening disease that poses significant challenges to the life of all family members (diagnosed child, parents, and siblings) and the family as a whole. To date, limited research has investigated family adjustment when facing pediatric cancer. The aim of the current study was to explore the role of protective factors at the individual (parental psychological flexibility), intrafamilial (dyadic coping) and contextual level (network support) in explaining family adjustment as perceived by parents of children with leukemia or non-Hodgkin lymphoma. In addition, we were interested to see whether these protective factors could be predictive for family adjustment at a later time point.

**Method:**

Participants were 70 mothers and 53 fathers (80 families) of children with leukemia or non-Hodgkin lymphoma. Mean time since diagnosis was 5.26 (T1) and 18.86 (T2) months post-diagnosis. Parents completed the Acceptance and Action Questionnaire II (to assess psychological flexibility), Dyadic Coping Inventory, a network support questionnaire, Impact on Family Scale and the Family Adjustment Scale. Both concurrent and prospective association models were tested.

**Results:**

Psychological flexibility, dyadic coping and network support proved to be cross-sectionally and positively related to parents’ perception of family adjustment post-diagnosis; psychological flexibility and dyadic coping proved to predict better family adjustment over time.

**Conclusion:**

Our findings led to the conclusion that protective factors at all three levels (individual, intrafamilial and contextual) are important for explaining family adjustment as perceived by parents facing a diagnosis of cancer in their child. Interventions targeting the individual, couple, as well as family level are warranted to enhance family adjustment.

## Introduction

Advances in the medical treatment of pediatric cancer have resulted in an increased survival rate and a shift in research focus from death and grief into the adjustment of children diagnosed with cancer and their family. Although pediatric cancer is a major stressor, the current body of literature on the adjustment of the diagnosed child, his/her parents and possible siblings suggest that most family members adjust well, and only a subset experiences psychosocial problems during or after treatment (individual adjustment). For example, symptoms of anxiety, depression (Brinkman et al., 2016) and distress (Michel et al., 2010) have been observed in some patients. Post-traumatic stress symptoms, emotional distress and anxiety are reported to a varying degree by parents (Grootenhuis and Last, 1997; Patino-Fernandez et al., 2008), and worry, loneliness, sadness and post-traumatic stress symptoms are reported by a subset of the siblings (Alderfer et al., 2010; Long et al., 2018).

In addition to the impact on the family members’ individual functioning, some studies have documented the impact of pediatric cancer on the family as a whole (family adjustment; see Pai et al., 2007; Van Schoors et al., 2015 for an overview). Overall, quantitative studies revealed that most families function within normative ranges (e.g., adaptability, Pai et al., 2007; family support, Brown et al., 2003) or even report improved functioning in some realms (e.g., cohesion, Cornman, 1993). Being on treatment and being a mother of a child with cancer, however, are risks factors for family conflict (Pai et al., 2007; Van Schoors et al., 2015). In contrast, qualitative studies into the family impact when facing pediatric cancer indicated a loss of normal family life (Koch, 1985; Clarke-Steffen, 1997; Bjork et al., 2009) and family rituals (Santos et al., 2018), troubles balancing multiple family needs including those of siblings (Bjork et al., 2009) and a shift in focus toward the diagnosed child at the cost of the family as a whole, the siblings and the couple relationship (Van Schoors et al., 2018a, b).

Given this variability in outcomes, both at the individual level and the family level (Kazak, 2006), a growing number of studies has tried to explain why some family members and families adjust better than others, investigating the role of potential protective factors. Based on existing research into pediatric oncology, protective factors that have been studied can be situated at three levels: the individual level (e.g., personality; Erickson and Steiner, 2001), the intrafamilial level (e.g., couple functioning; Santos et al., 2017; Van Schoors et al., 2019b) and the contextual level (e.g., network support; Corey et al., 2008).

To date, the current literature into individual and family adjustment when facing pediatric cancer is limited in four ways. First, most studies still tend to overlook outcomes at the family level (Van Schoors et al., 2015), and mainly focus on the consequences for individual family members facing pediatric cancer. Given the presumptions, however, that a family is more than the sum of its parts (Von Bertalanffy, 1973), and that a cancer diagnosis not only affects the individuals within the family, but also their relationships with one another and the way in which the family functions (Alderfer and Kazak, 2006), the family-level impact is undeniable. Second, most studies limit their scope to studying potential protective factors on one of the levels mentioned above (individual level, intrafamilial level, or contextual level). As a consequence, the existing studies mostly provide only a fragmented and partial explanation of the processes underlying post-diagnostic adjustment. This fragmented approach, however, is conceptually not in line with most contemporary family stress models (see Weber, 2011), who consider all these three categories of resources crucial to understanding the varying effects of external stressors on families, ranging from family crisis to family adjustment. According to these models, protective effects may reside in characteristics of individual patients and family members, characteristics of some of the family subsystems, as well as the broader context in which the family is embedded. Gaining further insight into the question why some families more effectively meet the demands of facing pediatric cancer than other, requires research that conceptually and empirically takes into account the multi-level nature of families’ resources. Third, most studies on adjustment after pediatric cancer relied on cross-sectional designs. As such, little is known on how family members/families adjust to the cancer diagnosis and its treatment over time. Fourth, within the pediatric cancer literature, most studies only include one single respondent (Van Schoors et al., 2015), rather than taking the perspectives of different family members into account, thereby neglecting the interdependence, and bidirectional relationships between different family members.

### The Present Study

In order to address these limitations, we conducted a study with two measurement points (T1 and T2) among parents (*mothers and fathers*) of children with leukemia or non-Hodgkin lymphoma to provide insight in the role of *individual, intrafamilial* and *contextual* protective factors for the family adjustment at T1 (first aim, cross-sectional), as well as to provide insight in the role of these factors *over time* on the family adjustment (second aim, from T1 to T2, prospective). Family adjustment was operationalized as the economic consequences for the family (financial impact), the disruption in the family’s normal social interactions (social impact), the disequilibrium experienced by the parents relating to the psychological burden of the illness (e.g., difficulty of planning for the future; general family impact) and the parents’ satisfaction with the family’s way of life (satisfaction with internal family fit). With respect to *individual protective factors* (=possessed by individual family members), this study examined the role of the child’s mother and father’s psychological flexibility. Psychological flexibility generally refers to the willingness of an individual to experience unwanted or aversive stressors while pursuing one’s values and goals, instead of avoiding unwanted or aversive stressors, thoughts and feelings (Hayes et al., 1999). Psychological flexibility has received scientific and clinical attention during recent years and showed to predict better well-being in patients and their caregivers (Kashdan and Rottenberg, 2010; Burke et al., 2014). With respect to *intrafamilial protective factors* (=possessed by the collective members of the family) that contribute to better outcomes in families facing pediatric cancer, this study examined the role of the couple’s dyadic coping. Dyadic coping refers to the extent to which partners deal with a stressor, like pediatric cancer, as a dyad (Bodenmann, 1995). Both theoretical (e.g., Systemic Transactional Model; Bodenmann et al., 2016) and empirical arguments (Badr et al., 2008) have illustrated the importance of couple variables, such as dyadic coping, within the context of health and illness-related issues. In addition, in a recent study (Van Schoors et al., 2019b), the importance of dyadic coping for the individual adjustment of parents facing leukemia or non-Hodgkin lymphoma in their child was illustrated. *Contextual protective factors* refer to the family’s social network (e.g., friends and relatives) and the support (e.g., emotional, informational) received from them. In the current study, the amount of perceived network support as reported by mothers and fathers, as well as discrepancies/congruencies between desired and received parental support (i.e., parental satisfaction with the received support) were included. Indeed, previous cancer research has showed that network support helps the family to better cope with the illness (Woodgate and Degner, 2003; Woodgate, 2006) and even reduces individual adjustment problems post-diagnosis (Hoekstra-Weebers et al., 2001).

Taken together, we expected that higher levels of psychological flexibility in mothers and fathers of children diagnosed with cancer (individual level), more adequate dyadic coping in their couple relationship (more stress communication, more supportive dyadic coping, more common dyadic coping, less negative dyadic coping; intrafamilial level) and more (amount and satisfaction with) support they receive from their network (contextual level) would be associated with better family adjustment (i.e., lower financial impact, social impact and general family impact, and more satisfaction with internal family fit), both cross-sectionally (at the same moment in time) and prospectively (after some time had passed).

## Materials and Methods

### Participants

The current sample consisted of 70 mothers and 53 fathers (80 families) where one child has been diagnosed with leukemia or non-Hodgkin lymphoma. All parents were Caucasian and living in the Flemish part of Belgium. The ill child’s mean age at diagnosis was 6.96 years (*SD* = 5.05; Range = 0–18). In 58 families (72.5%), the diagnosed child had been diagnosed with acute lymphoblastic leukemia (ALL). In the remaining families, 7 children (8.8%) had been diagnosed with acute myeloid leukemia (AML), one child (1.3%) with chronic myeloid leukemia (CML), and 14 children (17.5%) with Non-Hodgkin lymphoma. More details on the sample are listed in Table 1. Ethical approval from the University Hospitals of Ghent, Louvain, Brussels and Antwerp had been secured for the study and the appropriate written informed consent forms were obtained from all participants.

**TABLE 1:** Background characteristics of the study sample.

**Demographic variable**		
*N* (mothers, fathers)		123(70,53)
Age, mothers mean (*SD*)		37.61 (6.35)
Age, fathers mean *(SD)*		39.70 (6.41)
Age ill child at diagnosis, mean (*SD*)		6.96 (5.05)
Sex ill child, boys, *n (%)*		49(61.3%)
Diagnosis^1^, *n (%)*	ALL	58(72.5%)
	AML	7(8.8%)
	CML	1(1.3%)
	Non-Hodgkin Lymphoma	14(17.5%)
Time since diagnosis in months (*SD*, Range)	Time 1 (mothers)	4.74(5.87,0−28)
	Time 1 (fathers)	5.94(6.95,0−28)
	Time 2 (mothers)	19.46(11.51,3−45)
	Time 2 (fathers)	18.08(12.10,3−45)
Family status, *n (%)*	Married/Co-habiting	70(87.5%)
	Divorced	7(8.8%)
	Single parent	1(1.3%)
	Stepfamily	2(2.5%)
Number of children in the family, *n (%)*	One child	14(17.5%)
	Two children	36(45%)
	Three children	23(28.7%)
	Four children	5(6.3%)
	Five children	2(2.5%)

*Diagnosis^1^: ALL, acute lymphoblastic leukemia; AML, acute myeloid leukemia; CML, chronic myeloid leukemia.*

### Procedure

The present study is part of the “UGhent Families and Childhood Cancer Study,” a large study ongoing in Belgium, examining the impact of pediatric cancer on families (also see Van Schoors et al., 2019a, b). For this large-scale study, families of children diagnosed with leukemia or non-Hodgkin lymphoma (aged 0–18 years) were invited to take part in a survey study. The ill child (only when s/he was aged 5–18 years; younger patients did not complete questionnaires), their biological parents and any siblings (aged 5 years and more) were asked to complete a set of questionnaires at five different time points (from diagnosis to 2.5 years post-diagnosis). For the current study, parents with (at least) two measurements were included. If a parent had three or more measurements, his/her first and last measurement was taken into account. In the current study, mean time differences between measurement 1 (T1) and 2 (T2) were 15 and 12 months for mothers and fathers, respectively. Exclusion criteria for participation were: (1) not speaking Dutch, (2) relapse, and (3) the presence of a developmental disorder in the ill child. From the start of the study (September 2013), 137 families participated (65% of the eligible families); in 80 families at least two measurements per participant were available. The most important reasons for non-participation were lack of interest (41%), lack of time (27%) or being emotionally overwhelmed by the cancer (27%).

### Measures

#### Psychological Flexibility

The Acceptance and Action Questionnaire II (Bond et al., 2011) was used to assess parents’ ability to accept undesirable feelings and thoughts and to pursue their goals in the presence of potentially difficult experiences. The original questionnaire contains 10 items, rated on a 7-point Likert scale from 1 (*never true*) to 7 (*always true*) and is distributed across two factors. However, in accordance with Bond et al. (2011), the present study did not retain the three items on the second factor, as the predictive validity of the questionnaire was similar using a one-factor structure. All seven items were reversed, so higher scores indicate higher psychological flexibility. Total scores rage from 7 to 49. Example item are “Emotions cause problems in my life”, “Painful memories prevent me from having a satisfying life “and “I’m afraid of my feelings.” The AAQII has good reliability and validity (Bond et al., 2011). In the present study, Cronbach’s alpha coefficients were 0.89 and 0.93, for fathers and mothers, respectively.

#### Dyadic Coping

A short version of the Dyadic Coping Inventory (DCI; Bodenmann, 2008) was used to measure dyadic coping and stress communication. The questionnaire consists of 17 items, grouped into 6 subscales: Supportive Dyadic Coping (e.g., “S/he makes me feel that s/he understands me and is committed to me”; “S/he listens carefully and lets me speak, s/he responds appropriately to my stress or tries to lift me up”), Common Dyadic Coping (e.g., “We try to tackle the problem together and work together”; “We give each other emotional support”), Negative Dyadic Coping (e.g., “S/he does not take my stress seriously”; “S/he blames me for not being able to handle stress well”), Stress Communication (e.g., “When I feel overwrought, I show my partner that I feel bad and that I need his/her emotional support”), WE-Stress Appraisal (e.g., “If one of us is stressed, that is also “our” stress”) and Individual Stress-Appraisal (e.g., “If my partner is stressed, that’s his/her problem”). In the present study, (1) the two latter subscales were not included given our focus on dyadic coping strategies, and (2) the questionnaire was only completed by married or cohabiting parents. Response options for each item ranged from 1 to 5 (*very rarely* to *almost always*). Scores for the different subscales were obtained by summing the relevant items. The DCI has good reliability and validity (Ledermann et al., 2010). In the present study, Cronbach’s alpha coefficients were 0.72/0.78 (supportive dyadic coping), 0.91/0.93 (common dyadic coping), 0.69/0.81 (negative dyadic coping) and 0.85/0.91 (stress communication) for fathers and mothers, respectively.

#### Network Support

Our measurement of network support was based on the Psychosocial Assessment Tool (PAT; Kazak et al., 2001, 2015), a screening instrument designed to investigate psychosocial risk in families of children diagnosed with cancer. The PAT consists of 20 items, assessing a constellation of risk and resource factors, including social support (Kazak et al., 2001). For the present study, only the items relevant to network support were used. Participants had to indicate (*yes/no*) who they can count on to provide support, addressing six sources (Spouse/Partner, Patient’s Grandparents, Extended family, Friend, Work Associates, Other, None) and five forms of support (Childcare/Parenting, Emotional Support, Financial Support, Information, Help with everyday tasks). In addition, in accordance with the existing literature on helpful support (Rafaeli and Gleason, 2009), we also assessed the extent to which the support they received from their network is in line with what they need/desire. Answer options were *more than I need, exactly what I need* and *less than I need*. In the present study, two network support indices were included: (1) the total amount of perceived network support (i.e., sum across all sources of support and forms of support) (2) the satisfaction with the received network support (i.e., categorical variable; 3 levels).

#### Family Adjustment

The Impact on Family Scale (Stein and Riessman, 1980) and the Family Adjustment Scale (Antonovsky and Sourani, 1988) were used to assess the adjustment of the family system, as perceived by parents facing a pediatric cancer diagnosis in their child. The Impact on Family Scale (Stein and Riessman, 1980) consists of 33 items, distributed across 4 subscales: (1) Financial Burden (3 items; e.g., “The illness is causing financial problems for the family”), (2) Disruption of Social Relations (9 items; e.g., “We see family and friends less because of the illness”), (3) General Family Impact (19 items; e.g., “I don’t have much time left over for other family members after caring for my child”) and (4) Mastery (4 items; e.g., “Because of what we have shared we are a closer family”). All items were rated on a 4-point Likert scale from 1 (*strongly agree*) to 4 (*strongly disagree*). Subscales were calculated as the sum of all relevant (reverse scored) items, and a higher score indicated higher family impact, thus worse family adjustment. The questionnaire contained good validity and reliability (Stein and Riessman, 1980). In the present study, the Cronbach’s alpha coefficients were 0.66/0.68 (Financial Impact), 0.74/0.73 (Social Impact), 0.78/0.71 (General Family Impact) and 0.38/0.17 (Mastery), for fathers and mothers, respectively. Due to the low reliability of the latter subscale, this subscale was not included in the present study.

The Family Adjustment Scale (Antonovsky and Sourani, 1988) consists of 10 items and contains two subscales: satisfaction with internal family fit (8 items; e.g., “Are you satisfied with the family’s way of life?”) and family-community fit (2 items; e.g., “Are you satisfied with how your family fits into the neighborhood?”). Given our focus on family adjustment, the present study did not take into account the family-community fit subscale. All items are scored on a 7-point Likert scale from 1 (*totally unsatisfied*) to 7 (*totally satisfied*), with a higher score indicating higher satisfaction with internal family fit, thus better family adjustment. The FAS has good reliability (Antonovsky and Sourani, 1988). In the present study, Cronbach’s alpha coefficients were 0.94 (fathers) and 0.91 (mothers).

### Data Analytic Strategy

Reasons for selecting a multilevel modeling approach in the analysis of the data, rather than a single-level model, were twofold. First, the clustered sampling procedure (mothers and fathers from the same family) leads to non-independent observations: mothers and fathers from the same family tend to be more similar than mothers and fathers drawn at random from a population of parents. When using single-level methods (e.g., OLS multiple regression analysis) on non-independent data, standard errors tend to be underestimated. Such bias increases the rate of type I errors in statistical tests and may lead to incorrect statistical inference (Kenny and Judd, 1986). The multilevel approach, however, automatically adjusts for the effects of non-independent data and therefore more appropriate estimates of standard errors are obtained. Second, the multilevel approach enables us to address the relative contribution of individual and familial influences. The relative sizes of variance components at individual (i.e., individual characteristics of the parent, differences within families) and family level (i.e., family s/he belongs to, differences between families) provide information about the level at which the main processes operate.

Four dependent variables were tested: *financial, social and general family impact* as measured by the Impact on Family Scale and *satisfaction with internal family fit* as measured by the Family Adjustment Scale. The dependent variables were predicted by covariates (*time since diagnosis, age ill child, age parent, sex parent, diagnosis, family situation (i.e., married/cohabiting, divorced, single parent or stepfamily)* and the variables of interest *psychological flexibility* (AAQ-II; individual protective factor), *dyadic coping* (supportive, common, negative dyadic coping and stress communication; DCI; intrafamilial protective factor) and *network support* (total amount of perceived network support and satisfaction with received network support; contextual protective factor). Both concurrent and prospective association models were tested for each of the four dependent variables. First, in the concurrent association models, we evaluated how baseline levels of the predictors were associated with baseline levels of the dependent variables. This relationship is assessed in the following equation:

(1) $$Y_{i\,j}^{t\,1}=\beta_{0}+b_{i}$$

$$+\beta_{1}\,{\left(P\,s\,y\,c\,h\,o\,l\,o\,g\,i\,c\,a\,l\,f\,l\,e\,x\,i\,b\,i\,l\,i\,t\,y\right)}_{i\,j}^{t\,1}+\beta_{2}\,{\left(S\,u\,p\,p\,o\,r\,t\,i\,v\,e\,D\,C\right)}_{i\,j}^{t\,1}$$

$$+\beta_{3}\,{\left(C\,o\,m\,m\,o\,n\,D\,C\right)}_{i\,j}^{t\,1}+\beta_{4}\,{\left(N\,e\,g\,a\,t\,i\,v\,e\,D\,C\right)}_{i\,j}^{t\,1}$$

$$+\beta_{5}\,{\left(S\,t\,r\,e\,s\,s\,c\,o\,m\,m\,u\,n\,i\,c\,a\,t\,i\,o\,n\right)}_{i\,j}^{t\,1}+\beta_{6}\,{\left(T\,o\,t\,a\,l\,s\,o\,c\,i\,a\,l\,s\,u\,p\,p\,o\,r\,t\right)}_{i\,j}^{t\,1}$$

$$+\beta_{7}\,{\left(S\,a\,t\,i\,s\,f\,a\,c\,t\,i\,o\,n\,w\,i\,t\,h\,s\,o\,c\,i\,a\,l\,s\,u\,p\,p\,o\,r\,t\right)}_{i\,j}^{t\,1}$$

$$+\beta_{8}\,{\left(T\,i\,m\,e\,s\,i\,n\,c\,e\,d\,i\,a\,g\,n\,o\,s\,i\,s\right)}_{i\,j}^{t\,1}+\beta_{9}\,{\left(D\,i\,a\,g\,n\,o\,s\,i\,s\right)}_{i\,j}^{t\,1}$$

$$+\beta_{10}\,{\left(A\,g\,e\,i\,l\,l\,c\,h\,i\,l\,d\,a\,t\,d\,i\,a\,g\,n\,o\,s\,i\,s\right)}_{i\,j}^{t\,1}+\beta_{11}\,{\left(S\,e\,x\,p\,a\,r\,e\,n\,t\right)}_{i\,j}^{t\,1}$$

$$+\beta_{12}\,{\left(A\,g\,e\,p\,a\,r\,e\,n\,t\right)}_{i\,j}^{t\,1}+\beta_{13}\,{\left(F\,a\,m\,i\,l\,y\,s\,i\,t\,u\,a\,t\,i\,o\,n\right)}_{i\,j}^{t\,1}+\varepsilon_{i\,j}$$

where there are j observations for i families. b_i_ is the random effect with ${\text{b}}_{\text{i}}\,i.\text{i}.\text{d}.∼N\,\left(0,\sigma_{\text{b}}^{2}\right)$, allowing a different intercept for every family. In these models, the superscript t1 indicates that only observations of time 1 are included in the analyses. ε_ij_ is the within-family error component with ${\text{ε}}_{\text{ij}}\,i.\text{i}.\text{d}.∼N\,\left(0,\sigma_{\text{e}}^{2}\right)$.

Second, in the prospective association models, the dependent variables were predicted by the covariates and the variables of interest (as mentioned above), measured at the previous time-point. The time in between the two measurements varied between the participants from 1 to 32 months (*M* = 14, *SD* = 9) and was entered as an additional covariate. Time 2 measurements of the dependent variables were regressed on time 1 measurements of the predictors, following a blockwise hierarchical strategy. In the first block, covariates were entered along with time 1 status for each dependent variable to control for inherent stability. In the second block, the variables of interest were entered. We were interested in the amount of variance explained by the variables of interest that is not accounted for by previous status of the dependent variable. To formally test whether time 1 variables predicted the dependent variables at time 2 beyond initial status, we tested the statistical significance of the difference between block 1 (control for time 1 status) and block 2 (variables of interest) as indicated by the deviance statistic (−2^∗^LogLikelihood). The model equation used in the second block was:

(2) $$Y_{i\,j}^{t\,2}=\;\,\beta_{0}+b_{i}$$

$$+\beta_{1}\,{\left(P\,s\,y\,c\,h\,o\,l\,o\,g\,i\,c\,a\,l\,f\,l\,e\,x\,i\,b\,i\,l\,i\,t\,y\right)}_{i\,j}^{t\,1}+\beta_{2}\,{\left(S\,u\,p\,p\,o\,r\,t\,i\,v\,e\,D\,C\right)}_{i\,j}^{t\,1}$$

$$+\beta_{3}\,{\left(C\,o\,m\,m\,o\,n\,D\,C\right)}_{i\,j}^{t\,1}+\beta_{4}\,{\left(N\,e\,g\,a\,t\,i\,v\,e\,D\,C\right)}_{i\,j}^{t\,1}$$

$$+\beta_{5}\,{\left(S\,t\,r\,e\,s\,s\,c\,o\,m\,m\,u\,n\,i\,c\,a\,t\,i\,o\,n\right)}_{i\,j}^{t\,1}+\beta_{6}\,{\left(T\,o\,t\,a\,l\,s\,o\,c\,i\,a\,l\,s\,u\,p\,p\,o\,r\,t\right)}_{i\,j}^{t\,1}$$

$$+\beta_{7}\,{\left(S\,a\,t\,i\,s\,f\,a\,c\,t\,i\,o\,n\,w\,i\,t\,h\,s\,o\,c\,i\,a\,l\,s\,u\,p\,p\,o\,r\,t\right)}_{i\,j}^{t\,1}$$

$$+\beta_{8}\,{\left(T\,i\,m\,e\,s\,i\,n\,c\,e\,d\,i\,a\,g\,n\,o\,s\,i\,s\right)}_{i\,j}^{t\,1}+\beta_{9}\,{\left(D\,i\,a\,g\,n\,o\,s\,i\,s\right)}_{i\,j}^{t\,1}$$

$$+\beta_{10}\,{\left(A\,g\,e\,i\,l\,l\,c\,h\,i\,l\,d\,a\,t\,d\,i\,a\,g\,n\,o\,s\,i\,s\right)}_{i\,j}^{t\,1}$$

$$+\beta_{11}\,{\left(S\,e\,x\,p\,a\,r\,e\,n\,t\right)}_{i\,j}^{t\,1}+\beta_{12}\,{\left(A\,g\,e\,p\,a\,r\,e\,n\,t\right)}_{i\,j}^{t\,1}$$

$$+\beta_{13}\,{\left(F\,a\,m\,i\,l\,y\,s\,i\,t\,u\,a\,t\,i\,o\,n\right)}_{i\,j}^{t\,1}+\beta_{14}\,\left(Y_{i\,j}^{t\,1}\right)$$

$$+\beta_{15}\,{\left(T\,2-T\,1\right)}_{i\,j}^{t\,1}+\varepsilon_{i\,j}\,\text{i}$$

where there are j observations for i families. b_i_ is the random effect with ${\text{b}}_{\text{i}}\,i.\text{i}.\text{d}.∼N\,\left(0,\sigma_{\text{b}}^{2}\right)$, allowing a different intercept for every family. In these models, the outcome is taken at time 2 (superscript t2), while the predictors are taken at time 1 (superscript t1). The outcome at the previous time-point was included as a predictor in the model (${\text{Y}}_{\text{ij}}^{\text{t}\,1}$). ε_ij_ is the within-family error component with ${\text{ε}}_{\text{ij}}\,i.\text{i}.\text{d}.∼N\,\left(0,\sigma_{\text{e}}^{2}\right)$.

All multilevel analyses were performed with the R-package *lmerTest* (Kuznetsova et al., 2017). Equations for the models are given in Supplementary Equations S1, S2. Continuous predictors were grand mean centered in order to aid interpretation (Schielzeth, 2010). Models were fitted with restricted maximum likelihood (REML) estimation. The ANOVA table was inspected to check for significant effects and specific hypotheses were tested. Satterthwaite’s approximation was used to obtain the degrees of freedom (Sas Technical Report R-101, 1978). Model assumptions of linearity, independence, normality and homogeneity of variance were checked. The intra-class correlation coefficient (ICC) is reported as the amount of variance accounted for by differences between families rather than individual level components. For all statistical tests, significance levels were set at *p* < 0.05.

## Results

### Descriptive Analyses

Table 2 shows the descriptive statistics and correlations of the variables in the present study.

**TABLE 2:** Range, mean (*M*), standard deviation (*SD*) of the continuous variables of interest (psychological flexibility, dyadic coping and network support), and Pearson Correlation Coefficients between the two measurement points and between the variables of interest, aggregated over the two time-points.

	**Range**	**M (SD)**	**N**	**Cor (T1, T2)**	**1**	**2**	**3**	**4**	**5**	**6**
1. Psychological flexibility	7–49	35.30 (7.56)	123	0.71^∗^	–	0.16	0.35^∗^	−0.32^∗^	−0.01	0.15
Dyadic coping										
2. Supportive DC	4–20	13.35 (2.50)	104	0.64^∗^	–	–	0.73^∗^	−0.66^∗^	0.55^∗^	0.33^∗^
3. Common DC	3–15	12.20 (2.09)	104	0.55^∗^	–	–	–	−0.72^∗^	0.50^∗^	0.38^∗^
4. Negative DC	3–15	5.97 (2.11)	104	0.48^∗^	–	–	–	–	−0.31^∗^	−0.31^∗^
5. Stress communication	2–10	6.75 (1.76)	105	0.57^∗^	–	–	–	–	–	0.29^∗^
Network support										
6. Total support	0–30	14.23 (5.09)	123	0.75^∗^	–	–	–	–	–	–

*^∗^*p* < 0.05.*

### Concurrent Analyses

Regression coefficients and associated 95% confidence intervals (CI) for the models used are presented in Table 3.

**TABLE 3:** Cross-sectional model with variables measured at baseline.

**Predictor**	**Financial impact (*N* = 112, 75 families) coefficient B (CI)**	**Social impact (*N* = 112, 75 families) coefficient B (CI)**	**General family impact (*N* = 111, 74 families) coefficient B (CI)**	**Satisfaction with internal family fit (*N* = 112, 75 families) coefficient B (CI)**
**Variables of interest**				
Psychological flexibility	−0.02 (−0.07,0.04)	−0.07 (−0.16,0.03)	−0.11 (−0.21, −0.02)^∗^	0.29 (0.13,0.44)^∗∗∗^
Stress communication	0.15 (−0.08,0.38)	0.02 (−0.39,0.44)	0.27 (−0.13,0.68)	−0.06 (−0.70,0.59)
Supportive dyadic coping	−0.05 (−0.24,0.14)	−0.13 (−0.47,0.21)	−0.15 (−0.48,0.18)	0.44 (−0.10,0.97)
Common dyadic coping	0.02 (−0.24,0.27)	0.09 (−0.37,0.56)	0.03 (−0.42,0.49)	0.57 (−0.15,1.29)
Negative dyadic coping	−0.04 (−0.26,0.18)	−0.21 (−0.61,0.18)	−0.19 (−0.57,0.19)	−0.62 (−1.24, −0.01)^∗^
Total network support	−0.05 (−0.14,0.03)	−0.07 (−0.22,0.08)	−0.16 (−0.30, −0.01)^∗^	0.04 (−0.19,0.27)
Satisfaction with network support (too few vs. enough)	1.12 (−0.01,2.26)	0.83 (−1.25,2.91)	2.57 (0.48,4.66)^∗^	1.87 (−1.33,5.08)
Satisfaction with network support (too much vs. enough)	−0.35 (−1.54,0.83)	−0.67 (−2.84, 1.50)	−2.04 (−4.16,0.08)	0.87 (−2.48,4.22)
**Covariates**				
Time since diagnosis	0.002 (−0.06,0.07)	−0.10 (−0.22,0.03)	−0.13 (−0.25,−0.002)	−0.09 (−0.28,0.10)
Age ill child	−0.03 (−0.16,0.10)	−0.21 (−0.45,0.03)	−0.17 (−0.41,0.07)	−0.06 (−0.43,0.30)
Diagnosis^1^ (AML vs. ALL)	0.73 (−0.65,2.12)	−0.06 (−2.64,2.51)	1.39 (−1.16,3.94)	−0.07 (−3.98,3.83)
Diagnosis^1,2^ (CML vs. ALL)	−1.19 (−5.39,3.00)	−5.10 (−12.92,2.71)	−2.98 (−10.73,4.76)	15.52 (3.69,27.35)^∗^
Diagnosis^1^ (Non−Hodgkin vs. ALL)	−0.36 (−1.60,0.87)	0.51 (−1.79,2.81)	0.62 (−1.68,2.91)	0.60 (−2.88,4.08)
Sex parent (women vs. men)	0.51 (−0.30,1.33)	2.44 (0.98,3.90)^∗∗^	1.24 (−0.18,2.65)	0.44 (−1.87,2.76)
Age parent	−0.06 (−0.15,0.04)	−0.05 (−0.22,0.12)	−0.10 (−0.27,0.07)	0.05 (−0.21,0.31)
Family status (Step family vs. Married)	1.00 (−1.52,3.52)	−1.15 (−5.85,3.54)	−2.67 (−7.31,1.98)	−1.32 (−8.44,5.79)
Family status (Divorced vs. Married)	−0.10 (−3.11,2.92)	−0.80 (−6.34,4.74)	−2.19 (−7.65,3.26)	−15.29 (−23.81,−6.77)^∗∗∗^

*^∗^*p* < 0.05, ^∗∗^*p* < 0.01, and ^∗∗∗^*p* < 0.001.^1^ALL, acute lymphoblastic leukemia; AML, acute myeloid leukemia; CML, chronic myeloid leukemia. *^2^*Only 1 family with CML was included in the analysis.*

#### Financial Impact (IOF)

Thirty percent of the variance in the model could be explained by differences between families, and 70% was caused by individual level components. None of the predictor variables were significantly associated with financial impact (all *F* < 2.10, all *p* > *0.12*).

#### Social Impact (IOF)

Thirty six percent of the variance in the model could be explained by differences between families and 64% was caused by individual level components. Mothers reported more disruption of their social relations (higher social impact) than fathers [*F*(1,64.68) = 10.68, *p* = 0.002]. None of the other associations were significant (all *F* < 2.93, all *p* > *0.09*).

#### General Family Impact (IOF)

Forty percent of the variance in the model could be explained by differences between families, and 60% of the variance was caused by individual level components. More psychological flexibility was associated with less impact on the family, thus better family adjustment [*F*(1,92.72) = 5.18, *p* = 0.03]. In addition, higher levels of perceived network support was associated with less impact on the family, thus better family adjustment [*F*(1,92.80) = 4.35, *p* = 0.04]. Also, the satisfaction with the received support was of importance [*F*(2,92.23) = 4.77, *p* = 0.01]: parents receiving less support from their network than desired/needed (i.e., lower support satisfaction) showed a greater impact on the family, thus worse family adjustment, than those who reported to receive exactly the desired/needed amount of support (*p* = 0.02). Finally, the more time had passed since the diagnosis, the lower the impact on the family, thus the better the family adjustment [*F*(1,59.68) = 3.98, *p* = 0.05], but this association was only marginally significant. None of the other associations reached significance (all *F* < 2.94, *p* > 0.09).

#### Satisfaction With Internal Family Fit (FAS)

Twenty-nine percent of the variance in the model could be explained by differences between families and 71% was caused by individual level components. More psychological flexibility was associated with more satisfaction with internal family fit, thus better family adjustment [*F*(1,93.77) = 13.45, *p* < 0.001], whereas more negative dyadic coping was associated with less satisfaction with the internal family fit, thus worse family adjustment [*F*(1,83.48) = 3.99, *p* = 0.049]. Finally, the family situation was also of importance [*F*(2,61.44) = 6.24, *p* = 0.003]: divorced parents reported less satisfaction with internal family fit, thus worse family adjustment, than married or co-habiting parents (*p* < 0.001). There was no significant difference between stepfamilies and nuclear families (*p* = 0.14). None of the other associations was significant (all *F* < 2.22, *p* > 0.09).

### Prospective Analyses

Regression coefficients and associated 95% confidence intervals (CI) for the models used for the prospective analyses are presented in Supplementary Tables S1–S4.

#### Financial Impact (IOF)

There was a strong consistency for financial impact from time 1 to time 2 [*F*(1,92.12) = 43.29, *p* < 0.001]. Entry of the predictors of change improved the overall fit beyond that of time 1 status [χ^2^(8) = 24.83, *p* = 0.002]. There was a significant predictive effect of psychological flexibility [β = −0.08, 95% CI (−0.13,−0.03); *F*(1,87.93) = 10.22, *p* = 0.002]: higher levels of psychological flexibility at time 1 were predictive of lower financial impact at time 2. There was also a significant predictive effect of stress communication [β = −0.25, 95% CI (−0.46,−0.05); *F*(1,89.61) = 5.89, *p* = 0.02]: more stress communication at time 1 was predictive for a lower financial impact at time 2. None of the other variables had a significant predictive effect (all *F* < 3.05, *p* > 0.08).

#### Social Impact (IOF)

There was a strong consistency for social impact from time 1 to time 2 [*F*(1,97.63) = 15.28, *p* < 0.001]. Entry of the predictors of change did not significantly improve the overall fit beyond that of time 1 status [χ^2^(8) = 13.84, *p* = 0.09], and none of the variables of interest had a significant predictive effect (all *F* < 3.73, *p* > 0.05).

#### General Family Impact (IOF)

There was a strong consistency for general family impact from time 1 to time 2 [*F*(1,99.98) = 29.84, *p* < 0.001]. Entry of the predictors of change improved the overall fit beyond that of time 1 status [χ^2^(8) = 15.61, *p* = 0.048]. There was a significant predictive effect of psychological flexibility at time 1 [β = −0.16, 95% CI (−0.26, −0.06); *F*(1, 86.71) = 9.83, *p* = 0.002], indicating that higher levels of psychological flexibility at time 1 were predictive for a lower impact on the family, thus better family adjustment, at time 2. There was also a significant effect of time since diagnosis [β = −0.07, 95% CI (−0.14, −0.003); *F*(1,74.50) = 4.17, *p* = 0.045], indicating that the impact of the illness on the family was lower, thus better family adjustment, if more time had passed since diagnosis. None of the other variables had a significant predictive effect (all *F* < 2.62, *p* > 0.10).

#### Satisfaction With Internal Family Fit (FAS)

There was a strong consistency for satisfaction with internal family fit from time 1 to time 2 [*F*(1,97.97) = 21.18, *p* < 0.001]. Entry of the predictors of change did not significantly improve the overall fit beyond that of time 1 status [χ^2^(8) = 9.75, *p* = 0.28], and none of the variables of interest had a significant predictive effect (all *F* < 2.61, *p* > 0.11).

## Discussion

The aim of the current study was to explore the role of potential protective factors at the individual (psychological flexibility), intrafamily (dyadic coping), and contextual level (network support) in explaining family adjustment (i.e., financial impact, social impact, general family impact, satisfaction with internal family fit) as perceived by parents of children with leukemia or Non-Hodgkin lymphoma. By taking into account protective factors at all three levels, we aimed to explain the existing variability in family outcomes when facing pediatric cancer. In addition, we investigated whether this variance was explained by individual and/or familial components; as well as the stability/changes in family adjustment across time.

### Summary of Results

#### Psychological Flexibility and Family Adjustment

Our findings indicate that psychological flexibility, defined as an individual (here, the parents) willingness to experience unwanted or aversive stressors while pursuing one’s values and goals (Hayes et al., 1999), is important for the family adjustment as perceived by parents facing leukemia/Non-Hodgkin lymphoma in their child, both cross-sectionally and prospectively. This is in line with our prediction and with previous research on psychological flexibility in parents of children with cancer (Burke et al., 2014). However, different patterns of findings emerged for general and financial family consequences.

More specifically, we found that, both concurrently and prospectively, more psychological flexibility in parents was associated with a lower general impact on the family and, concurrently, higher satisfaction with internal family fit. In other words, the more a parent “accepts” his/her negative thoughts and emotions, the better the family adjustment, both concurrently and prospectively. This finding is in line with the idea that psychological flexibility is an important protective factor in predicting individual adjustment outcomes (Kashdan and Rottenberg, 2010). In addition, in the context of cancer, these negative thoughts and emotions may be centered around the illness and its treatment. Indeed, when facing pediatric cancer, psychological flexibility may refer to a sense of acceptance of the diagnosis or the transition to be a cancer patient or to have a child with cancer, as well as the acceptance of the uncontrollable and possibly fatal nature of the illness. This “acceptance” has been shown to improve individual psychosocial outcomes in patients with cancer (Carver et al., 1993; Stanton et al., 2002; Hulbert-Williams et al., 2015) and parents of children with cancer (Burke et al., 2014).

Moreover, the present study extends previous research on psychological flexibility, as – to the best of our knowledge – it is the first investigating the association between psychological flexibility and *family* adjustment instead of individual adjustment. Based on existing literature, we know that pediatric cancer often causes parental distress post-diagnosis (i.e., anxiety, depression, post-traumatic stress symptoms, Grootenhuis and Last, 1997; Patino-Fernandez et al., 2008), and that psychological flexibility can operate as a buffer for parental maladjustment (individual level; Burke et al., 2014). As parents play a cardinal role in their family, and parental functioning is linked to the way in which the family as a whole functions (theoretical argument: Social Ecology Model, Bronfenbrenner, 1977; empirical argument: Kashdan et al., 2004), we might assume that the underlying mechanism underneath the association between psychological flexibility and family adjustment may be the parents’ individual functioning: the more parents accept their negative thoughts and emotions, the better their individual functioning (e.g., less anxiety; depression) and therefore the better the adjustment of the family as a whole. More research is needed, however, to confirm this hypothesis.

In addition, there was also a significant prospective association between psychological flexibility and the financial impact in families being confronted with pediatric cancer. So, the more parents “accept” their negative thoughts and emotions in the short term, the less they are worried about the financial consequences of the illness in the long term. It is possible that accepting negative thoughts/emotions in general, and cancer-related thoughts/emotions in specific, helps parents to accept the financial impact as well, as these parents may potentially focus more on the well-documented “positive side-effects” of the cancer diagnosis (e.g., increased closeness within the family; Van Schoors et al., 2015, 2018a).

#### Dyadic Coping and Family Adjustment

Our findings indicate that dyadic coping can be linked to the adjustment of the family as perceived by parents being confronted with a cancer diagnosis in their child, both cross-sectionally and prospectively. Specifically, we found that more stress communication predicted a smaller financial impact in families being confronted with pediatric cancer (prospective finding). In other words, the more mothers and fathers shared their stress with their partner in the short term, the less they were worried about the financial consequences of the illness in the long term. Explanations are twofold. First, it is plausible to assume that couples sharing their illness-related stress, also share other worries, e.g. financial worries. As social sharing reduces stress (Rimé, 1995), we might assume that – although the objective financial impact stayed the same – the parental concerns about the financial consequences might decline with increased stress communication. Second, stress communication can be seen as a characteristic of “expressiveness” As a consequence, a parent sharing stress with his/her partner is likely to share stress with others (e.g., friends, grandparents of the diagnosed child) as well. When others know about possible financial problems in the family of the diagnosed child, they can help by, for example, giving/borrowing money or organizing benefits. This explanation is strengthened by the present data: we found a significant correlation of 0.27 between stress communication and the total amount of perceived network support.

Furthermore, there was an association between negative dyadic coping and satisfaction with internal family fit (cross-sectional finding). The more a parent experiences distancing, mocking or sarcasm from his/her partner when talking about the illness, the worse the perceived family adjustment. This is in line with previous studies investigating the association between negative dyadic coping and negative (individual) outcomes in adult chronically ill populations (Meier et al., 2011) and parents of children with cancer (Van Schoors et al., 2019b). Surprisingly, however, there was no significant association between positive dyadic coping (supportive dyadic coping and common dyadic coping) and family adjustment. Explanations are twofold. First, the absence of a significant association between positive dyadic coping and family adjustment could be due to limited statistical power. Second, this finding may also suggest that positive dyadic coping is particularly important for the individual adjustment of parents being confronted with pediatric cancer (as previously found by Van Schoors et al., 2019b), and not the family adjustment. However, more research is needed to confirm this hypothesis.

#### Network Support and Family Adjustment

Our findings indicate that network support is important for the family adjustment as perceived by parents of children with cancer (cross-sectional finding). More specifically, we found that higher levels of network support as perceived by parents were related to a lower general impact on the family, thus better family adjustment. This finding emphasizes the importance of network support when facing pediatric cancer (Hoekstra-Weebers et al., 2001; Woodgate and Degner, 2003; Woodgate, 2006). In addition, when taking into account discrepancies and congruencies between desired and received parental support, we found that when parents received the exact amount of support they needed/desired, they reported a lower family impact, and thus better family adjustment, as compared to parents receiving less support than needed/desired. To note, no significant differences were found between parents receiving the exact amount of support and parents receiving more support than needed/desired. This is in contrast to some studies (e.g., Siewert et al., 2011) showing “the more, the better”; i.e., that the overprovision of support is related to higher well-being.

#### Other Findings

The results of the present study furthermore revealed the importance of *sex, time since diagnosis* and the *family situation* in the prediction of family adjustment. First, mothers reported a higher social impact of their child’s illness than fathers (cross-sectional finding). Indeed, in most of the included families the mother (temporally) has quit her job to ensure that always one parent could accompany the diagnosed child to the hospital, whereas the father kept working to ensure financial security. As a consequence, whereas the mother’s daily life changed completely, the father’s daily activities stayed more or less the same as pre-diagnosis (Van Schoors et al., 2018b). Second, parents living in a family with a child who has been diagnosed more recently reported worse family adjustment than those who had been exposed to the illness for a more prolonged period of time, both cross-sectionally and prospectively. This can be linked to the treatment course of the cancer, and the intensity of the hospitalizations needed to cure the child. Whereas at diagnosis, intense treatment with long hospitalizations are needed, these hospitalizations decrease over time with only 1-day care treatment after some months/a year. As especially being separated as a family (mother and ill child in the hospital vs. father and siblings at home) is hard to handle for the different family members (Van Schoors et al., 2018a, b), decreased hospitalizations of the ill child can be linked to more time together as one family, and thus better family adjustment. Third, divorced parents reported a lower satisfaction with internal family fit, thus worse family adjustment, than married or cohabiting parents (cross-sectional finding). We might assume that working together as a team (mother and father) helps a parent to cope with the cancer diagnosis, and therefore helps the family as a whole to fulfill all family needs (e.g., individual needs of all family members including those of siblings, household needs, and financial needs) (Van Schoors et al., 2018b), whereas divorced parents are mostly obliged to manage the cancer situation alone. This explanation is strengthened by the present study’s finding that the family adjustment is comparable for nuclear families and stepfamilies, emphasizing the need to divide family tasks in order to keep their head up in these difficult times.

Furthermore, for all outcomes of interest (financial impact, social impact, general family impact, satisfaction with internal family fit) both individual characteristics and the differences between families seem to be important. In other words, in predicting family adjustment, researchers should take into account *who* (individual characteristics of the parent) is reporting, as well as the *family* s/he belongs to. This is in line with a recent study on the individual adjustment of family members (patients, mothers, fathers, and siblings) facing pediatric cancer (Van Schoors et al., 2019a).

Finally, the present study found that family adjustment at time 1 was an important predictor for family adjustment at time 2. This indicates that the relative adjustment of families compared to other families remains stable: families who score relatively high at time 1 will still score relatively high at time 2 and vice versa. In addition, within each family, the adjustment improves over time as evidenced by additional analyses that included *time* as predictor variable (general impact: β_*time*_ = −1.07, *p* = 0.004; financial impact: β_*time*_ = −0.39, *p* = 0.03; social: β_*time*_ = −1.85, *p* < 0.001; satisfaction with internal family fit: β_*time*_ = −1.72, *p* = 0.03). This is in line with existing quantitative literature showing that although – over time – families return to “normal” again (van Buiren et al., 1998), discrepancies between families occur in the adaptation process post-diagnosis. Indeed, the Pediatric Psychosocial Preventative Health Model (PPPHM; Kazak, 2006) divides families of children in pediatric health care settings into three groups: (1) the so-called *Universal* group, which is the largest group and consisting of families showing at least moderately resiliency and possessing adequate to strong coping abilities, (2) the *Targeted* group includes those families at higher risk and in need of some services and (3) the *Clinical/Treatment* group refers to families at highest risk; showing more evident symptomatology. In order to facilitate bon-adjustment post-diagnosis, family needs should be matched with clinical services.

### Strengths and Limitations

A first strength of the present study is the design. By taken into account two measurements, we were able to examine the temporal order of the associations under investigation. Second, although most studies in the pediatric cancer literature make use of a single-family member participant (Van Schoors et al., 2015), we included the perspectives of both parents. Third, protective factors at all three levels (individual level, intrafamilial level and contextual level) were included in the present study, whereas previous research mostly focused on only one of these levels. As a consequence, to the contrary of most existing studies who provided only a fragmented explanation of the processes underlying post-diagnostic family adjustment, we were able to present a more complete picture of factors fostering adjustment in families facing pediatric cancer.

Despite the strengths of the study, there were also some limitations. A first limitation is the small sample size. With only 70 mothers and 53 fathers, we can only draw limited conclusions regarding the association between psychological flexibility, dyadic coping, network support and family adjustment, as perceived by these parents. In addition, given the small sample size and the high number of included variables, non-significant results could also be due to limited power. Further research, with larger samples, are therefore needed to confirm our findings. Second, our sample consisted of Caucasian, heterosexual couples, thereby limiting the generalizability of our results. Future research should attempt to replicate these findings with more heterogeneous samples, e.g., homosexual couples. In addition, the Dutch language was an inclusion criterion for participation in the study. With respect to the current multicultural society, however, this language criterion might have been a barrier for ethnic minorities. Third, we only focused on families with leukemia or non-Hodgkin lymphoma in one of the children. It is important to highlight that parents of children with other cancer diagnoses, e.g., brain tumors, may have different experiences. Fourth, mean time since diagnosis was 5.26 (T1) and 18.86 (T2) months post-diagnosis. In order to best capture the adaptation process post diagnosis, however, the first measurement should be as close as possible to the moment of diagnosis. In addition, researchers should include comparison groups (e.g., families with healthy children; families with a suspicion of pediatric cancer but no actual diagnosis, families where one child is diagnosed with a brain tumor), so more information about cancer specific processes vs. processes that are similar across specific health-related conditions can be explored. Fifth, the mean age of the patients in the present study was 6.96 years. Most of the patients were toddlers and primary school children. Further research with families of adolescents and young adults with cancer (AYA’s) is needed, as the developmental stage of the children may indeed influence factors important for family functioning. Sixth, although we included predictive variables at all three levels (individual, intrafamily and contextual level), only one variable per level was selected. Further research should investigate other predictive variables, at all three levels. Finally, as one of the main reasons for non-participation was being emotionally overwhelmed by the diagnosis, we might assume that especially more resilient families participated in our study (selection bias). Other findings might occur for emotionally distressed families.

### Clinical Implications

Our findings provide evidence that a pediatric cancer diagnosis not only impacts the individual functioning of the different family members, but also the family functioning. Three specific recommendations arise from the study findings. First, clinical interventions should be tailored to gender differences and specific characteristics of mothers and fathers facing pediatric cancer. Indeed, our findings suggest that mothers might be in greater need of psychosocial support, as they perceived the social disruption post-diagnosis as more severe. Second, across our findings, especially the association between psychological flexibility (individual protective factor) and family adjustment seems to be important. As a consequence, families could in particular benefit from interventions targeting the promotion of acceptance of unwanted negative thoughts and emotions, e.g., using Cognitive Behavioral Therapy or Acceptance and Commitment Therapy (Hayes et al., 2012). Third, when facing pediatric cancer, a holistic approach – including individual, couple and family interventions – is needed to best help families to cope with this severe stressor. Indeed, the findings of the present study showed that protective factors at all three levels (individual, intrafamilial, and contextual level) are important for the adjustment of the family as a whole. Moreover, as family adjustment is both explained by individual characteristics (“who filled in the questionnaire?”) and differences between families (“the family s/he belongs to), both the individuality of each family members, as well as the mutual and bidirectional influences within families should be taken into account by clinicians.

## Data Availability Statement

The raw data supporting the conclusions of this article will be made available by the authors, without undue reservation, to any qualified researcher.

## Ethics Statement

The study involving human participants was reviewed and approved by the Ethical Committee from the University Hospitals of Ghent, Louvain, Brussels, and Antwerp. The patients/participants provided their written informed consent to participate in this study.

## Author Contributions

All authors read and reviewed the manuscript, and contributed to it in a meaningful way. MV wrote the manuscript, under the supervision of LV and LG. AD did the statistical analysis. JL, AM, KN, and KL helped in particular with the clinical implications outlined in the manuscript.

## Conflict of Interest

The authors declare that the research was conducted in the absence of any commercial or financial relationships that could be construed as a potential conflict of interest.
