# Supplementary material for: Family Adjustment When Facing Pediatric Cancer: The Role of Parental Psychological Flexibility, Dyadic Coping, and Network Support
Source: Front Psychol. 2019 Dec 10;10:2740. doi: 10.3389/fpsyg.2019.02740 (PMC6914864; doi:10.3389/fpsyg.2019.02740)
Supplement: Supplementary file 1 [file Table_1.pdf]

Supplementary Equation S1

For the concurrent association models, this was the general equation:

$$Y_{ij}^{t1} = \beta_0 + b_i + \beta_1 X_{ij}^{t1} + \cdots + \varepsilon_{ij}$$

There are j family members for i families. b_i is the random effect with b_i $\mathrm{i.i.d.} \sim \mathrm{N}\left(0, \sigma_\mathrm{b}^2\right)$, allowing a different intercept for every family. In these models, the superscript t1 indicates that only observations of time 1 are included in the analyses. ε_{ij} is the within-family error component with ε_{ij} $i.i.d. \sim N(0, \sigma_e^2)$.

Supplementary Equation S2

For the prospective association models, this was the general equation:

$$Y_{ij}^{t2} = \beta_0 + b_i + \beta_1 X_{ij}^{t1} + \beta_2 Y_{ij}^{t1} + \cdots + \varepsilon_{ij}$$

There are j family members for i families. b_i is the random effect with b_i $\mathrm{i.i.d.} \sim \mathrm{N}\left(0, \sigma_\mathrm{b}^2\right)$, allowing a different intercept for every family. In these models, the outcome is taken at time 2 (superscript t2), while the predictors are taken at time 1 (superscript t1). The outcome at the previous time-point was included as a predictor in the model ($\mathrm{Y}_\mathrm{ij}^\mathrm{t1}$) . ε_{ij} is the within-family error component with ε_{ij} $i.i.d. \sim N(0, \sigma_e^2)$.

Table S1. *Models fit for the prospective analyses of the dependent variable General family impact*.

	Block 1: Control for initial status	Block 2 Adding variables of interest
Predictor	General family impact T2 (N = 111, 74 families) *Coefficient B [CI]*	General family impact T2 (N = 111, 74 families) *Coefficient B [CI]*
Variables of interest		
Psychological flexibility T1	-	-.16 [-.26, -.06]**
Stress communication T1	-	.03 [-.40, .46]
Supportive DC T1	-	.04 [-.30, .38]
Common DC T1	-	.24 [-.23, .71]
Negative DC T1	-	-.04 [-.43, .34]
Total network support T1	-	-.07 [-.23, .08]
Satisfaction with network support (too few vs. enough) T1	-	.94 [-1.27, 3.15]
Satisfaction with network support (too much vs. enough) T1	-	.43 [-1.83, 2.69]
Covariates		
Time since diagnosis	-.07 [-.14, .006]	-.07 [-.14, -.003]*
Age ill child	-.09 [-.33, .16]	-.07 [-.32, .18]
Diagnosis (AML vs. ALL)	.74 [-2.08, 3.55]	.80 [-2.02, 3.64]
Diagnosis (CML vs. ALL)	-3.78 [-11.27, 3.71]	-4.96 [-12.50, 2.58]
Diagnosis (Non Hodgkin vs. ALL)	.40 [-2.17, 2.98]	.30 [-2.27, 2.87]
Sex parent (women vs. men)	.91 [-.35, 2.17]	.55 [-.91, 2.01]
Age parent	.002 [-.17, .17]	-.01 [-.19, .16]
Family status (Divorced vs. Married)	2.69 [-.84, 6.21]	2.47 [-1.19, 6.12]
T2 minus T1	-.07 [-.14, .005]	-.06 [-.14, .01]
**Outcome variables at previous time**		
Financial impact T1	-	-
General family impact T1	.52 [.33, .70]***	.38 [.17, .59]***
Social impact T1	-	-
Satisfaction with internal family fit T1	-	-
**Δ Deviance[1]**	29.06***	15.61*

To note: ALL = Acute lymphoblastic leukemia, AML = Acute myeloid leukemia, CML = Chronic myeloid leukemia; [1]For the control model (block 1), the deviance is relative to the model with only covariates. For the prediction model (block 2) , the deviance is relative to the control model; * p < .05, ** p < .01, *** p < .001

Table S2. *Models fit for the prospective analyses of the dependent variable Financial impact*.

	Block 1<br>Control for initial status	Block 2<br>Adding variables of interest
Predictor	Financial impact T2<br>(N = 111, 74 families)<br>*Coefficient B [CI]*	Financial impact T2<br>(N = 111, 74 families)<br>*Coefficient B [CI]*
Variables of interest		
Psychological flexibility T1	-	-.08 [-.13, -.03]**
Stress communication T1	-	-.26 [-.46, -.05]*
Supportive DC T1	-	.16 [-.02, .33]
Common DC T1	-	.11 [-.12, .35]
Negative DC T1	-	-.04 [-.23, .16]
Total network support T1	-	-.03 [-.10, .04]
Satisfaction with network support (too few vs. enough) T1	-	.25 [-.78, 1.29
Satisfaction with network support (too much vs. enough) T1	-	.44 [-.61, 1.49]
Covariates		
Time since diagnosis	-.004 [-.04, .03]	-.007 [-.04, .02]
Age ill child	.02 [-.09, .13]	.01 [-.10, .12]
Diagnosis (AML vs. ALL)	.16 [-1.09, 1.41]	.16 [-.1.02, 1.33]
Diagnosis (CML vs. ALL)	-2.80 [-6.04, .44]	-3.82 [-6.86, -.79]*
Diagnosis (Non Hodgkin vs. ALL)	-.58 [-1.73, .57]	-.52 [-1.58, .55]
Sex parent (women vs. men)	-.02 [-.72, .68]	.07 [-.73, .88]
Age parent	.02 [-.06, .10]	.03 [-.05, .11]
Family status (Divorced vs. Married)	.31 [-1.36, 1.97]	.91 [-.70, 2.51]
T2 minus T1	-.002 [-.04, .04]	-.01 [-.05, .03]
**Outcome variables at previous time**		
Financial impact T1	.62 [.43, .80]***	.57 [.39, .75]***
General family impact T1	-	-
Social impact T1	-	-
Satisfaction with internal family fit T1	-	-
**Δ Deviance[1]**	39.25***	24.83**

To note: ALL = Acute lymphoblastic leukemia, AML = Acute myeloid leukemia, CML = Chronic myeloid leukemia; [1]For the control model (block 1), the deviance is relative to the model with only covariates. For the prediction model (block 2) , the deviance is relative to the control model; * p < .05, ** p < .01, *** p < .001

Table S3. *Models fit for the prospective analysis of the dependent variable Social impact.*

	Block 1<br>Control for initial status	Block 2<br>Adding variables of interest
Predictor	Social impact T2<br>(N = 111, 74 families)<br>*Coefficient B [CI]*	Social impact T2<br>(N = 111, 74 families)<br>*Coefficient B [CI]*
Variables of interest		
Psychological flexibility T1	-	-.10 [-.19, .001]
Stress communication T1	-	.01 [-.40, .43]
Supportive DC T1	-	.12 [-.20, .45]
Common DC T1	-	.31 [-.15, .77]
Negative DC T1	-	-.01 [-.38, .36]
Total network support T1	-	-.08 [-.23, .06]
Satisfaction with network support (too few vs. enough) T1	-	1.05 [-1.05, 3.15]
Satisfaction with network support (too much vs. enough) T1	-	1.03 [-1.13, 3.19]
Covariates		
Time since diagnosis	-.06 [-.13, .008]	-.06 [-.13, .01]
Age ill child	-.07 [-.31, .17]	-.05 [-.30, .20]
Diagnosis (AML vs. ALL)	2.03 [-.69, 4.75]	2.00 [-.80, 4.80]
Diagnosis (CML vs. ALL)	-5.14 [-12.45, 2.18]	-6.56 [-14.17, 1.04]
Diagnosis (Non Hodgkin vs. ALL)	-.31 [-2.81, 2.20]	-.63 [-3.19, 1.94]
Sex parent (women vs. men)	.14 [-1.11, 1.38]	.05 [-1.40, 1.51]
Age parent	-.005 [-.17, .16]	-.01 [-.18, .16]
Family status (Divorced vs. Married)	2.15 [-1.29, 5.58]	2.40 [-1.20, 6.00]
T2 minus T1	-.05 [-.13, .02]	-.04 [-.11, .04]
**Outcome variables at previous time**		
Financial impact T1	-	
General family impact T1	-	
Social impact T1	.41 [.21, .61]***	.34 [.14, .54]**
Satisfaction with internal family fit T1	-	
**Δ Deviance[1]**	15.81***	13.84

To note: ALL = Acute lymphoblastic leukemia, AML = Acute myeloid leukemia, CML = Chronic myeloid leukemia; [1]For the control model (block 1), the deviance is relative to the model with only covariates. For the prediction model (block 2) , the deviance is relative to the control model; * $p < .05$, ** $p < .01$, *** $p < .001$

Table S4. *Models fit for the prospective analysis of the dependent variable Satisfaction with internal family fit.*

	Block 1<br>Control for initial status	Block 2<br>Adding variables of interest
Predictor	Satisfaction with internal family fit T2<br>(N = 109, 73 families)<br>*Coefficient B [CI]*	Satisfaction with internal family fit T2<br>(N = 109, 73 families)<br>*Coefficient B [CI]*
Variables of interest		
Psychological flexibility T1	-	.16 [-.07, .39]
Stress communication T1	-	-.15 [-1.08, .79]
Supportive DC T1	-	-.24 [-1.02, .54]
Common DC T1	-	.46 [-.59, 1.52]
Negative DC T1	-	-.39 [-1.25, .48]
Total network support T1	-	.21 [-.13, .54]
Satisfaction with network support (too few vs. enough) T1	-	-1.38 [-6.10, 3.34]
Satisfaction with network support (too much vs. enough) T1	-	.20 [-4.62, 5.02]
Covariates		
Time since diagnosis	.09 [-.06, .25]	.10 [-.06, .25]
Age ill child	.37 [-.13, .87]	.22 [-.31, .76]
Diagnosis (AML vs. ALL)	2.55 [-3.37, 8.47]	2.85 [-3.13, 8.82]
Diagnosis (CML vs. ALL)	12.64 [-3.51, 28.79]	17.80 [1.11, 34.49]*
Diagnosis (Non Hodgkin vs. ALL)	.39 [-4.98, 5.76]	.31 [-5.09, 5.72]
Sex parent (women vs. men)	.98 [-1.65, 3.61]	2.47 [-.79, 5.73]
Age parent	-.35 [-.70, .01]	-.24 [-.62, .14]
Family status (Divorced vs. Married)	-6.92 [-15.68, 1.84]	-7.43 [-16.45, 1.60]
T2 minus T1	.06 [-.10, .23]	.08 [-.10, .26]
**Outcome variables at previous time**		
Financial impact T1	-	-
General family impact T1	-	-
Social impact T1	-	-
Satisfaction with internal family fit T1	.50 [.29, .72]***	.30 [.01, .59]*
**Δ Deviance[1]**	21.25***	9.75

To note: ALL = Acute lymphoblastic leukemia, AML = Acute myeloid leukemia, CML = Chronic myeloid leukemia; [1]For the control model (block 1), the deviance is relative to the model with only covariates. For the prediction model (block 2) , the deviance is relative to the control model; * p < .05, ** p < .01, *** p < .001
